# Kidney Transplantation Is Associated with Catastrophic Out of Pocket Expenditure in India

**DOI:** 10.1371/journal.pone.0067812

**Published:** 2013-07-04

**Authors:** Raja Ramachandran, Vivekanand Jha

**Affiliations:** 1 Department of Nephrology, Postgraduate Institute of Medical Education and Research, Chandigarh, India; 2 George Institute for Global Health, New Delhi, India; University of Colorado, United States of America

## Abstract

Kidney transplantation (KT) is only viable renal replacement option for most patients in India. Most patients do not have health insurance and meet treatment expenditure from their own resources. We prospectively evaluated the expenses associated with KT and its impact on the socioeconomic status of families in a public hospital. All direct and indirect expenses incurred by the patients from the time of diagnosis of chronic kidney disease to KT were recorded. Direct expenses included physician fees, cost of drugs and disposables, dialysis, and expenses on investigations and hospitalization. Indirect expenses included travel, food, stay, and loss of income suffered by the family. Educational dropout and financial loss were also recorded. There were 43 males and 7 females between the ages of 12 and 57 years. Direct expenses ranged from US$ 2,151–23,792 and accounted for two-thirds of the total expenses. Pre-referral hospitalization, dialysis and medication accounted for majority of direct expense. Indirect expenses ranged from US$ 226–15,283. Travel expenses and loss of income accounted for most of indirect expense. About 54%, 8%, and 10% of families suffered from severe, moderate, and some financial crisis respectively. A total of 38 families had job losses, and 1 patient and 12 caregivers dropped out of studies. To conclude, KT is associated with catastrophic out-of-pocket expenditure and pushes a majority of the patients who come for treatment to public hospitals into severe financial crisis. Educational dropout and loss of jobs are other major concerns. Systematic efforts are required to address these issues.

## Introduction

Around the world, the cost incurred on treatment of end-stage renal disease (ESRD) is an important contributor to national health-related expenditure [1], [2]. The total healthcare spending ESRD in US was $ 32.9 billion in 2010 [3]. Of all forms of renal replacement therapy (RRT), kidney transplantation is the least expensive.

The age-adjusted incidence of ESRD in India is estimated at 226 per million population [4]. As hemodialysis is not widely available, living related donor transplantation soon after the diagnosis is the only viable form of long-term RRT for most patients.

Reimbursement for healthcare is available only to a minority. In the absence of state or private insurance schemes, most citizens, especially those who are self-employed or work in unorganized sector, have to make out-of-pocket (OOP) expenses to meet healthcare-associated costs. Only the wealthy can afford treatment in private hospitals. The poor typically seek treatment in public sector hospitals where the government subsidizes treatment. They do not have to pay for medical advice, procedures, and investigations but must pay for drugs and disposables. Long queues for limited number of slots force patients to get dialysis in private centers while awaiting transplantation [5]. Transplant centers are distributed unevenly throughout the country. Lack of a system of referral forces patients to travel long distances, often with multiple family members to seek out transplant facilities [6]. This entails expenses on travel, food and housing. Loss of job and interruption of education of patients and family members are additional sources of revenue loss.

It has been suggested that ESRD treatment imposes a major financial burden on citizens in poor countries [5]. Multiple studies and meta-analysis have evaluated the indirect cost incurred by living kidney donors [7], but none of the studies have evaluated the indirect cost and consequence incurred by the renal transplant recipient and their family. Consequently, the economic burden of treatment in Indian ESRD subjects is unknown. We conducted this study to determine direct and indirect expenses associated with subsidized kidney transplantation in a public sector hospital in India and assess its impact on the socioeconomic status of families of the affected.

## Materials and Methods

### Ethics Statement

The study protocol was approved by the Postgraduate Institute of Medical Education and Research (PGIMER) Institute Ethics Committee, and all subjects provided written consent. In the case of the sole minor subject, the father provided written consent.

This prospective observational study was done on 50 consecutive patients who presented to the Nehru Hospital, PGIMER, Chandigarh for getting a kidney transplant between March and September 2011. This is a major public sector tertiary referral hospital with a referral base over north India, and has a large transplant program (about 200/year).

Demographic data including age, gender, address, cause of kidney disease, time since diagnosis, occupation, monthly income, and source of funding were recorded. Also recorded were preexisting or newly discovered comorbidities.

According to hospital policy, patients with household incomes below poverty line defined by the Government are entitled to waiver of hospital charges. Several patients receive assistance from government or private charities to meet with part of treatment cost. This information was recorded from official documents.

Data on direct and indirect expenses incurred by the patient from the time of diagnosis till undergoing renal transplant were collected. Patients were asked to preserve all the receipts and where these were not available, to record the expenditure in a diary.

### Direct Expenses

This was defined as the amount spent by the patient on physician fees, investigations, hospitalization, dialysis, drugs and disposables. All expenses were documented directly from the receipts.

### Indirect Expenses

The monies spent by patient and family members on travel, accommodation, food (above the usual amount spent in normal health) and loss of income suffered by the patients and family members were categorized under indirect expenses. Patients and caregivers provided documentation of expenses. Where such documents were not available (e.g., food, local travel), a reasonable estimate based on documentation in the diary was accepted. Loss of income was calculated by multiplying the weekly income with the number of weeks the person was unemployed.

In most cases, patients had visited private nephrologists and/or hospitals before presenting to our Institution. In most instances, a clear break up of direct and indirect expenses was not available. Since treatment in private hospitals is expensive and likely to constitute bulk of the expenses, all expenses before coming to our Institution were classified as direct expenses.

The extent of financial loss, loss of work and instances of patients/dependents discontinuing education were also recorded. Financial loss was categorized as severe, moderate, some or none. Severe loss was defined as total loss of all property and loss of occupation; moderate financial loss was loss of property without loss of occupation/source of income, or loss of occupation with loss of some property; some financial loss as loss of some property without any occupational loss; and no loss meant no loss of any property and no loss of job. Education dropout meant discontinuation of studies due to dislocation or financial loss, or in order to find work to support the family.

Finally, on the day before the surgery, patients were asked the following questions:

1. Before coming here, did you know how much would you need to spend on transplantation?
2. Do you regret the decision to go ahead with transplantation because of financial reasons?
3. If you had to do it again, will you still go ahead?
4. Based on your experience, will you advise others in your situation to get a transplant?
5. Do you have an identified source of funding your future immunosuppressive therapy?

All costs were calculated per patient basis. Data are presented as mean or median with an appropriate measure of dispersion. The total sum of each component of direct and indirect expenses of all 50 patients was calculated using GraphPad prism software. Correlation was examined between presence and degree of catastrophic expenditure and patients characteristics (age, gender, marital status), and socioeconomic (distance between place of residence and transplant center, level of income and education, employment status, source of funding) and clinical variables (cause of ESRD and time since diagnosis).

## Results

Of the fifty subjects included in the study, there were 43 (86%) males and 7 (14%) females with ages ranging from 12 to 57 years. A majority of our patients presented within 12 months of diagnosis of end stage renal failure.

Twenty-five (50%) of the patients were self-employed, 6 were salaried workers, 6 (12%) were homemakers, 3 (6%) were students, 4 were unemployed and 1 was a businessman. The monthly household income ranged from US$ 38–943. Thirty-six (72%) patients were the sole bread-earners for their families.

Patients had traveled from 10–2,600 km in order to get a transplant. In all cases, the travel was by public transport (bus or train). Patients were accompanied by 2–10 (median 4) caregivers. All caregivers were related to patients and hence no expense was incurred on social assistance.

### Source of Funding

**Figure 1:**
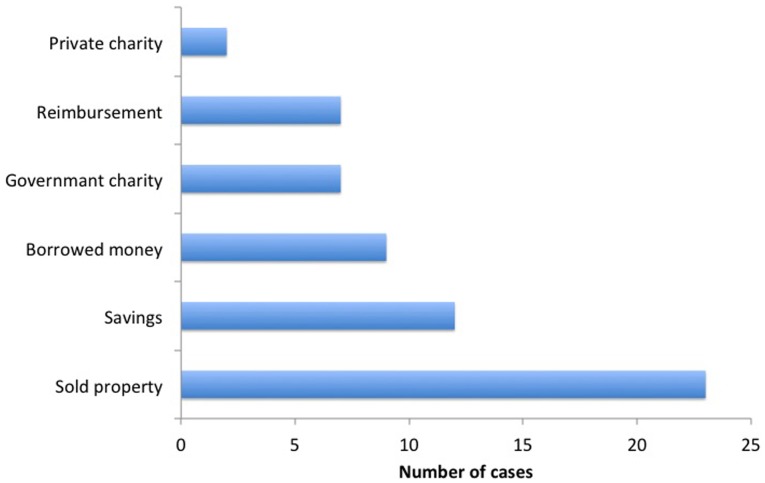
Showing the source of funding (the total exceeds 50 as some patients had more than one funding source).

Fig. 1 shows the source of funding for the treatment-related expenditure. A total of 31 (62%) patients had documentary proof of “below poverty line” status and thus were exempt from paying hospital charges. Expenses were borne by the patients in 28 (56%) cases, whereas for 22 (44%), the cost of treatment was borne by another family member. Seven (14%) patients received assistance amounting to 50,000–150,000 Indian rupees (943–2830 U.S. dollars) from government charities.

### Expenses

A breakup of the direct and indirect expenses for the patients is provided in Tables 1 and 2. Direct expenses ranged from US$ 2,151 to 23,792. Pre-referral hospitalization expense (US$ 2,186±3,234) was the most important contributor to direct expenses followed by expenses on the day of transplant and the cost of hospitalization during transplant work-up (US$ 0–3,773). Only 20% patients needed hospitalization after referral. The cost incurred on dialysis was almost equivalent to the hospitalization expenses, largely because all patients had to undergo dialysis for varying periods in private centers. Indirect expenses ranged from US$ 226–15,283. Most of the indirect expenses were due to loss of income of the patient and caregivers. Food, stay and transport also added a significant amount into the indirect expenses [Table 2]. Direct expenses constituted 67.2% of total expenditure.

**Table 1:** Direct expenses of renal transplant.

	Mean ± SD	Range	Median	Interquartile range
Dialysis	2,230±2,432	0–10,868	1,364	671–2,887
Medicine	1,443±1,463	59–7,925	975.5	557–1,843
Investigations	337±398	19–2,830	283	127–472
Hospitalization				
Before referral	2,186±3,234	0–18,868	972	377–2,830
After referral	285±825.3	0–3,774	0	0–0
Transplant expenditure	1,400±376	1,038–2,925	1,321	1,226–1,415
**Total**	**7,881±5,308**	**2,151–23,792**	**6,240**	**4,148–9,771**

All values in US$.

**Table 2:** Indirect expenses.

	Mean ± SD	Range	Median	Interquartile range
Transport	650±834	45–3774	317	175–698
Housing	355±348	0–1358	297	0–566
Food	506±455	0–1585	396	83–792
**Income Loss**				
Caregiver	986±1605	0–6792	415	0–1132
Patient	1364±1897	0–7925	764	0–1981
Total	**3862**±**2995**	**226–15283**	**3094**	**1618–5647**

All values in US$.

**Figure 2:**
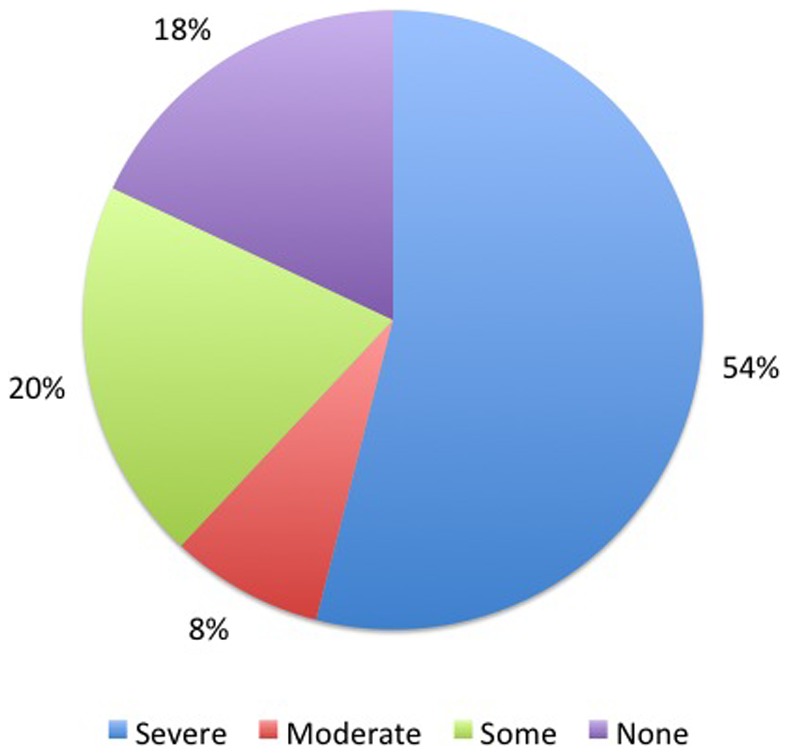
Pie chart shows the extent of financial crisis suffered by the families of the patients.

A total of 41 (82%) patients experienced financial crisis during the course of treatment [Figure 2]. The crisis was severe for 27 (54%) patients, moderate for 4 (8%), and some for 10 (20%) patients. Of the 8 patients who had access to reimbursement, 2 patients had some financial loss and 6 had no financial loss. Twenty-eight (56%) patients and 10 (20%) caregivers lost their jobs/livelihood as a result of the illness. One (2%) patient and 12 (24%) dependents/caregivers discontinued their education directly as a result of this illness.

Two (4%) patients required inpatient care prior to renal transplant for respiratory tract infections, for 15 days and 3 months respectively. Four (8%) patients tested positive for hepatitis C virus while undergoing HD in outside units. All of them had been negative at initial work up and were presumed to have acquired the infection in the dialysis units. None of them had resources to undergo treatment for hepatitis C prior to renal transplant, and chose to go ahead with transplantation. Three (6%) of them developed transient transaminitis, which delayed the surgery in one by 7 weeks. File S1 provides patient demographics and type of expenses: direct and indirect; out-of-pocket expenditures and household incomes from all cases.

Of the various parameters examined, the following correlated significantly with the degree of catastrophic OOP expenditure younger age (p = 0.0002), male gender (p = 0.002), lower education level (p = 0.03), higher hospitalization expenses before referral (p = 0.02), lack of full-time employment (p = 0.0004) and non-availability of medical reimbursement (<0.0001).

Table 3 shows the results of the questionnaire administered on the day before transplantation. It shows that the patients were largely positive and happy with their decision to go ahead with transplantation despite the crippling expenses. However, they might have not have made the same decision if they had full knowledge of the consequences. Also, their advice to others in this situation would be different. Crucially, a majority of patients did not have identified source of funding the post-transplant immunosuppression.

**Table 3:** Responses to the questions asked the day before surgery.

Question	Number (%) responding yes
1. Compared to your initial estimate, was the amount of money you ended up spending more?	13 (26)
2. Looking back, do you regret the decision to go ahead with transplantation because of financial reasons?	10 (20)
3. If you had to do it again, will you go ahead?	13 (26)
4. Will you advise others in your financial situation to get a transplant?	14 (28)
5. Do you have an identified source for funding long-term immunosuppressive therapy	11 (22)

A few illustrative case summaries are provided in File S2.

## Discussion

ESRD has been described as a devastating condition for patients and families [8]. A large proportion of ESRD patients in India either does not start or discontinues RRT due to financial reasons [9], [10]. The cost-effectiveness of kidney transplantation over dialysis is well-documented [11], [12]. The present study shows that the families of a large proportion of those who get even this cheapest form of RRT in a public sector hospital suffer major economic setbacks, which must be weighed against the benefits of treatment. This is the first study to systematically evaluate the cost of getting a kidney transplant in India. The patient population described in this study is fairly representative of the families in the lower-middle and low socioeconomic groups in India in terms of income and employment.

As shown in this study, the cost of getting kidney transplantation in a public sector hospital in India is far less than that described almost anywhere else in the world. This, however, needs to be placed in context of the overall socioeconomic environment. According to the 2010 World Bank Data, the per capita gross national product (GNP) of India based on purchasing power parity is US$ 3,590, and despite a large number with growing income, about 33% still live on US$1.25 a day [13].

The high out-of-pocket OOP expenditures are not a surprise. India does not have a clear-cut healthcare reimbursement policy. The current government spending on health is 0.9% of GDP, among the lowest in the world [14]. Private expenditure on health is close to 78%, compared to 14% in the Maldives, 29% in Bhutan, 53% in Sri Lanka, 31% in Thailand and 61% in China. According to one estimate, only about 5.5% of the Indian population has some form of medical insurance [14]. Practically none of the families below poverty line have any healthcare coverage. Indian healthcare expenses are borne by households in 73.5% cases, by the public sector in 22% and 4.5% from other sources [15].

The factors associated with the degree and severity of financial crisis are predictable. Of note are the association with male gender and younger age, reflecting the affliction of the primary wage-earner of the family. Also worth noting is the association with the interval between time of diagnosis and referral, leading to higher pre-referral expenditure and greater financial crisis.

Need for OOP financing leaves the Indian population exposed to risk of unforeseen expenditures. According to our definition, 54% of patients in this study experienced severe financial crisis. Catastrophic health expenditure is defined as one that reduces the non-health care expenditure to a level where the household is unable to maintain consumption of necessities [16]. The threshold for such expenditure has been arbitrarily set at 10% of the total household budget [17]–[19], or 40% of non-food expenditure [20]. In our study, these thresholds were crossed by a large margin. Had we taken any of the two cutoffs, the frequency of families with catastrophic expenditure would be over 90%. Interestingly, two patients who had access to reimbursement also experienced some crisis. This is because of the policy of patients having to initially pay for treatment and claim reimbursement later, which requires initial OOP spending.

The scope of the current study covered only the period till the surgery. Despite not being aware of the expenses and having suffered significant hardships, a majority of patients did not regret the decision to have come thus far, because they could not find any alternative. However, they were less sanguine in their answer to the other questions, viz. whether they would do it again or advise others in the same situation to do so. Superficially these responses seem contradictory but perhaps reflect the reality that they discovered during the course of treatment. Also alarming was the fact that 78% had no clear idea of how to finance post-transplant immunosuppression. Most were hoping to get some assistance from charities. If this does not come through or after it runs out, the only option is to sell any leftover assets or borrow money further. In addition, the future of family members of more than half of the subjects has been jeopardized directly by treatment-related expenses. In case they are unable to continue treatment, the purpose of a sophisticated treatment like transplant will be defeated. The cycle of ever increasing loan repayments and reduced earning ability is likely to push families further into poverty.

Absence of identifiable treatment coverage plan or payer source has been considered an absolute contraindication to transplantation [21]. In a situation where patients fund their own treatment, the treating physician/surgeon must depend upon the patients’ own assessment of their ability to meet with treatment costs. Going ahead with transplantation is often an emotional decision, and many subjects were unaware of the magnitude of expense. Once started, they found it hard to go back on the process. Having a sick member forces financially constrained families to choose between cutting household consumption and going without treatment.

Xu et al identified three preconditions for catastrophic expenditure, viz. the availability of health services requiring payment, low capacity to pay, and the lack of prepayment or health insurance, all of which exist in India [22]. Public sector hospitals like ours provide a partial safety net by subsidizing inpatient care, but still leave patients exposed to heavy OOP expenditure for outpatient treatment including drugs and ambulatory care (like dialysis). In some cases, the hospital helps them in securing funds through charities but these are unorganized and unpredictable.

Indirect expenses constituted one-third of the total costs. Since all expenses before the referral were classified under direct due to unavailability of breakup of direct and indirect components, this is likely an underestimate of the actual indirect cost. Loss of the caregiver’s income was the largest contributor to the indirect cost followed by transport expenses and loss of income of the patient. Again, as this study stops at the time of transplantation, the ability of these people to get re-employed and rehabilitated in a competitive job market is unknown and will lead to continued financial hardships.

The reported case studies show drastic changes in the expenditure patterns and lifestyle changes for families of affected individuals. With the sole focus on meeting treatment costs, expenditure on all aspects of life for the entire family was severely curtailed. Sacrifice of education is a matter of particular concern. Education is the key to socioeconomic success. This not only reduces the opportunity of the affected to optimally utilize their potential, but also worsens the overall level of education of the country, leading to a loss to the society.

Prevention of development and progression of chronic kidney disease (CKD) has rightly received attention over the last decade. Despite this, the number of patients who need dialysis and renal transplantation are likely to rise. According to the Indian CKD registry, about 48% patients receive a diagnosis of CKD for the first time in stage 5 [23], and hospital data show that over 70% patients require dialysis soon after presentation [10]. At our hospital, the number of kidney transplants every year rose from about 100 to about 200 over the last 3 years.

Since the numbers are likely to rise, two important questions must be answered: first, whether renal transplantation should be offered to Indian ESRD patients without any concrete financial plan for treatment, and second, to what extent should the government subsidize this process. Some states (eg. Tamil Nadu, Andhra Pradesh and Goa) have started schemes that provide free transplant and immunosuppression [24]. However, the coverage of such schemes is still poor. Experience from countries like Thailand, Mexico and some Latin American countries suggests that expansion of universal coverage can limit the OOP expenditure on RRT [25], [26]. However, this requires allocation of a large budget. In our study too, availability of reimbursement reduced the likelihood of suffering financial losses.

Even within the available resources, a number of strategies can reduce the impoverishing burden of the OOP expenditure. These include pre-emptive transplantation [27], increased use of peritoneal dialysis as a bridge to transplantation [25], avoidance of use of more expensive immunosuppressive drugs like mycophenolate mofetil in favor of cheaper ones like azathioprine and use of metabolic inhibitors to reduce the dose requirement of calcineurin inhibitors [28]. Insurance should not involve pre-payment by the patient. In addition to strategies to reduce direct treatment costs, ways need to be devised to reduce the indirect costs as well. This requires increasing the number of transplant centers in the public sector hospitals and making their distribution uniform across the country, as this will reduce the need to travel long distances, prevent family dislocation and reduce the likelihood of loss of jobs and discontinuation of education.

Some limitations of our study must be acknowledged. We evaluated expenditure pattern in only those households that actually presented for treatment, and did not take into account families who have a patient with ESRD could not afford treatment. Many patients make a decision not to continue with treatment because of insufficient resources. This triaging disproportionately affects the disadvantaged, such as women and children. This is reflected in the low proportion of women in our study. Our study was a cross-sectional quantitative study limited till the time of transplant. Qualitative studies with longitudinal design are needed to estimate the long-term consequences of expenditure for post-transplant care on households. It will also be important to determine the extent to which people enrolled in some health insurance schemes are still exposed to OOP and catastrophic expenditures. This was a single center study and does not capture the range of expenditure that patients might incur in different hospitals. Moreover, the study was conducted in a public sector hospital where many services are free to the poor, the degree of expenditure is likely to be more elsewhere. Finally, the findings of this study are applicable to other diseases that involve sudden expenditure, but the long-term need for immunosuppression makes transplantation particularly a long-term financial burden for families.

### Conclusions

This is the first study of direct as well as indirect expenses incurred by families of ESRD patients undergoing renal transplantation in a public sector hospital in India and shows that such treatment is associated with catastrophic healthcare expenditure. Indirect expenses contribute least one-third of expenses, and renal transplantation pushes majority of the families into severe poverty. Of particular concerns are the loss of jobs and causes education dropouts in family members.

## Supporting Information

File S1 **Showing details of income and expenditure of all cases.**
(DOCX)

File S2 **Illustrative Case Summaries.**
(DOCX)
