# Supplementary material for: Kidney Transplantation Is Associated with Catastrophic Out of Pocket Expenditure in India
Source: PLoS One. 2013 Jul 4;8(7):e67812. doi: 10.1371/journal.pone.0067812 (PMC3701634; doi:10.1371/journal.pone.0067812)
Supplement: File S1 — Showing details of income and expenditure of all cases. (DOCX) [file pone.0067812.s001.docx]

| **Table: Showing details of income and expenditure of all cases** | | | | | | | | | | | | | | | | | |
| --- | --- | --- | --- | --- | --- | --- | --- | --- | --- | --- | --- | --- | --- | --- | --- | --- | --- |
| **Age** | **Gender** | **Occupation** | **Monthly family income** | **Finances** | **Dialysis** | **Drugs and disposables** | **Investigations** | **Pre-referral hospitalization** | **Hospitalization after referral** | **Direct expenses** | **Transport** | **Housing** | **Food** | **Income loss (caregivers)** | **Income loss (self)** | **Indirect expenses** | **Financial crisis** |
| 31 | M | Coal mine worker | 377 | Reimbursement+ sold land | 340 | 1340 | 472 | 0 | 0 | 2151 | 60 | 415 | 415 | 0 | 1509 | 2400 | some |
| 35 | M | Shop worker | 38 | Sold property+Govt grants+relatives contributed | 30 | 1382 | 94 | 981 | 0 | 2488 | 132 | 906 | 1509 | 566 | 943 | 4057 | Severe |
| 21 | M | Umemployed | 38 | Has borrowed money | 0 | 2132 | 19 | 377 | 0 | 2528 | 189 | 0 | 51 | 0 | 0 | 240 | Severe |
| 30 | M | Owns business | 189 | Sold property | 679 | 1380 | 132 | 717 | 0 | 2908 | 151 | 311 | 340 | 1698 | 1132 | 3632 | Moderate |
| 31 | F | Clerk | 75 | Reimbursement | 453 | 2109 | 377 | 0 | 0 | 2940 | 423 | 0 | 0 | 0 | 2717 | 3140 | None |
| 35 | M | Farmer | 94 | Borrowed money from friends | 679 | 1687 | 94 | 943 | 0 | 3404 | 83 | 170 | 283 | 566 | 283 | 1385 | Severe |
| 44 | M | Peon | 47 | Sold wife's ornaments | 679 | 2494 | 189 | 94 | 0 | 3457 | 102 | 283 | 566 | 0 | 283 | 1234 | Severe |
| 41 | M | Supervisor | 302 | Reimbursement | 623 | 1519 | 566 | 755 | 0 | 3462 | 788 | 340 | 566 | 453 | 1811 | 3958 | None |
| 22 | M | Farmer | 75 | Sold property | 255 | 1683 | 57 | 1132 | 377 | 3504 | 321 | 0 | 0 | 377 | 302 | 1000 | Severe |
| 38 | F | Homemaker | 113 | Saved money by brothers | 1019 | 1853 | 283 | 755 | 0 | 3909 | 2102 | 491 | 283 | 1019 | 0 | 3894 | some |
| 45 | F | Homemaker | 943 | Savings | 1340 | 2309 | 283 | 189 | 0 | 4121 | 226 | 0 | 0 | 0 | 0 | 226 | None |
| 34 | F | Homemaker | 377 | Father's pension | 1389 | 1920 | 472 | 358 | 0 | 4139 | 340 | 566 | 660 | 1132 | 0 | 2698 | None |
| 41 | M | Farmer | 113 | Sold property | 906 | 2113 | 283 | 189 | 660 | 4151 | 151 | 472 | 566 | 0 | 906 | 2094 | Severe |
| 28 | F | Homemaker | 283 | Financed by the family | 1811 | 2140 | 47 | 472 | 0 | 4470 | 196 | 755 | 755 | 4528 | 0 | 6234 | some |
| 24 | M | Carpenter | 57 | Father's saving | 906 | 1936 | 189 | 1509 | 0 | 4540 | 438 | 226 | 377 | 226 | 226 | 1494 | Severe |
| 55 | M | Clerk | 189 | Reimbursement | 1887 | 1792 | 396 | 472 | 0 | 4547 | 177 | 189 | 377 | 0 | 0 | 743 | None |
| 41 | M | Lorry driver | 283 | Sold land | 906 | 2306 | 226 | 1132 | 0 | 4570 | 98 | 792 | 906 | 906 | 1698 | 4400 | Severe |
| 57 | M | Teacher | 377 | Reimbursement | 849 | 3481 | 472 | 0 | 0 | 4802 | 574 | 679 | 1132 | 5660 | 0 | 8046 | None |
| 26 | F | Housewife | 151 | Loan from friends+ Govt relief fund | 1691 | 2249 | 283 | 660 | 0 | 4883 | 3774 | 0 | 0 | 0 | 0 | 3774 | Severe |
| 31 | M | Contracter | 377 | Loan | 453 | 2019 | 189 | 2264 | 0 | 4925 | 283 | 283 | 377 | 660 | 0 | 1604 | Moderate |
| 28 | M | Shop worker | 94 | Govt grants+ Other NGOs+ loan | 1721 | 2502 | 38 | 943 | 0 | 5204 | 386 | 0 | 934 | 1038 | 1038 | 3396 | Severe |
| 37 | M | Elecrtician | 226 | Sold property | 1766 | 2709 | 94 | 755 | 0 | 5325 | 189 | 528 | 1057 | 0 | 5434 | 7208 | Severe |
| 20 | M | Student | 132 | Sold property | 566 | 2509 | 189 | 2358 | 0 | 5623 | 108 | 453 | 528 | 849 | 0 | 1938 | Severe |
| 30 | M | Tea stall worker | 94 | Father's and brother's savings | 417 | 4219 | 453 | 943 | 0 | 6032 | 925 | 57 | 283 | 0 | 377 | 1642 | Severe |
| 46 | M | Buisness | 377 | Sold property | 725 | 3389 | 377 | 1604 | 0 | 6094 | 170 | 340 | 604 | 0 | 2264 | 3377 | some |
| 26 | M | Shop owner | 283 | Govt fund+ self finances | 1698 | 1423 | 94 | 3170 | 0 | 6385 | 543 | 453 | 566 | 2547 | 2547 | 6657 | Severe |
| 44 | M | Policeman | 566 | Govt Reimbersement | 1132 | 4226 | 472 | 377 | 377 | 6585 | 1509 | 0 | 0 | 0 | 0 | 1509 | some |
| 42 | M | Tailor | 189 | Savings | 2717 | 3106 | 472 | 415 | 0 | 6709 | 755 | 0 | 113 | 0 | 2264 | 3132 | some |
| 39 | M | Rice broker | 151 | Loan | 1155 | 3826 | 189 | 1755 | 0 | 6925 | 566 | 0 | 0 | 660 | 396 | 1623 | Moderate |
| 22 | M | Unemployed | 283 | Sold tractor | 443 | 2520 | 94 | 4321 | 0 | 7378 | 57 | 453 | 226 | 1887 | 0 | 2623 | Severe |
| 12 | M | Student | 377 | Loan | 75 | 1771 | 377 | 5283 | 0 | 7507 | 283 | 136 | 94 | 0 | 0 | 513 | Severe |
| 34 | M | Works at spinning mill | 340 | Saved money+ Govt grants+sold his ancestral land+ Father's savings | 4075 | 1940 | 472 | 1698 | 0 | 8185 | 423 | 519 | 943 | 0 | 6113 | 7998 | Severe |
| 37 | M | Teacher | 472 | Sold property | 645 | 1830 | 226 | 3774 | 1887 | 8362 | 434 | 396 | 396 | 6792 | 1887 | 9906 | some |
| 39 | M | Bus driver | 75 | Sold property | 3623 | 2702 | 189 | 1887 | 0 | 8400 | 1811 | 0 | 0 | 283 | 906 | 3000 | Severe |
| 39 | M | Farmer | 283 | Sold property | 2717 | 5264 | 377 | 377 | 0 | 8736 | 2830 | 566 | 566 | 4528 | 6792 | 15283 | some |
| 23 | M | Tailor | 66 | Govt assistance + self finances | 2008 | 2879 | 189 | 2830 | 943 | 8849 | 211 | 142 | 396 | 925 | 1321 | 2994 | Severe |
| 47 | M | Farmer | 94 | Sold property | 5434 | 2358 | 472 | 1132 | 0 | 9396 | 609 | 132 | 132 | 0 | 1132 | 2006 | Severe |
| 39 | M | insurance agent | 189 | Relatives | 3679 | 4849 | 94 | 962 | 113 | 9698 | 362 | 736 | 1132 | 0 | 7925 | 10155 | None |
| 38 | M | Carpenter | 132 | Sold property | 4075 | 3557 | 472 | 1887 | 0 | 9991 | 679 | 0 | 0 | 679 | 2377 | 3736 | Severe |
| 52 | M | Clerk | 943 | Insurance | 2594 | 2566 | 283 | 4717 | 0 | 10160 | 1132 | 0 | 0 | 0 | 0 | 1132 | None |
| 22 | M | Shop worker | 85 | Sold property | 2491 | 2002 | 113 | 5660 | 0 | 10266 | 136 | 0 | 0 | 1321 | 849 | 2306 | Severe |
| 25 | M | Student | 283 | Govt grants+ Other NGOs | 3532 | 3555 | 377 | 3491 | 0 | 10955 | 313 | 943 | 755 | 5094 | 0 | 7106 | some |
| 43 | M | Newspaper editor | 189 | Govt grants | 7943 | 3312 | 434 | 377 | 0 | 12066 | 219 | 830 | 1245 | 1509 | 3585 | 7389 | some |
| 43 | M | Temple worker | 113 | Sponsored by temple | 7472 | 6509 | 472 | 0 | 0 | 14453 | 2830 | 0 | 226 | 0 | 0 | 3057 | Moderate |
| 21 | M | Welder | 57 | Sold shop | 4075 | 5883 | 113 | 2830 | 1887 | 14789 | 226 | 906 | 1585 | 1698 | 1019 | 5434 | Severe |
| 39 | M | Teacher | 377 | Sold property | 3057 | 3019 | 755 | 7547 | 3774 | 18151 | 283 | 1155 | 1019 | 566 | 3396 | 6419 | Severe |
| 46 | M | Driver | 170 | Sold property | 9962 | 3400 | 283 | 5094 | 0 | 18740 | 45 | 594 | 1528 | 0 | 4075 | 6243 | Severe |
| 28 | M | Unemployed | 189 | Sold property | 2830 | 3325 | 566 | 10849 | 3774 | 21344 | 1149 | 1358 | 1132 | 1132 | 679 | 5451 | Severe |
| 29 | M | Unemployed | 189 | Sold property | 1087 | 1940 | 189 | 18868 | 0 | 22083 | 185 | 192 | 679 | 0 | 0 | 1057 | Severe |
| 38 | F | Homemaker | 755 | Reimbursement | 10868 | 9245 | 2830 | 377 | 472 | 23792 | 2536 | 0 | 0 | 0 | 0 | 2536 | None |
