# Supplementary material for: Kidney Transplantation Is Associated with Catastrophic Out of Pocket Expenditure in India
Source: PLoS One. 2013 Jul 4;8(7):e67812. doi: 10.1371/journal.pone.0067812 (PMC3701634; doi:10.1371/journal.pone.0067812)
Supplement: File S2 — Illustrative Case Summaries. (DOCX) [file pone.0067812.s002.docx]

**Case Summaries**

1. A 21 year-old married welder (monthly income US$ 62) was diagnosed with end stage renal disease (ESRD) at a private hospital. He was taking hemodialysis once every 10 days and at a cost of US$ 38 per dialysis. Over time, he developed severe malnutrition, wasting and neuropathy, which required repeated hospitalizations and intensification of dialysis at a private hospital. To arrange finances for his treatment, he sold his wielding shop for about US$ 8,000. This amount was exhausted and he had to borrow money from his relatives at a high interest rate. At this time, he came to our hospital for a kidney transplant accompanied by his family (parents, wife and son). They lived together in a hired room, received free food from the hospital canteen and *langar* (community kitchen organized in temples). The hospital helped him get money to fund his transplant surgery from government “relief funds”, and provided subsidized dialysis, (US$ 5/session). After surgery, he was started on treatment with generic tacrolimus-based therapy along with ketoconazole. The immunosuppressive drug were provided free of cost for the first 6 months as part of a study. Subsequently, he is able to fund his treatment with donations. He is now 1-year post transplant on tacrolimus, ketoconazole, azathioprine and steroids.
2. A twenty-eight old daily-wage worker in a tea-stall (monthly salary US$ 85) was diagnosed with ESRD 3 months back. He had no assets that could be sold to fund treatment. He received US$ 2,000 as donations from NGOs and travelled 1,500 kms for a getting a transplant to our hospital with family (parents and wife). By that time, he had already spent most of the assistance and taken loan from local moneylender. With assistance from the hospital, he received a special grant of about US$ 2,850 from the Prime Minister’s Relief Fund. In order to save money, patient’s family ate one meal a day. In the process, the parents became malnourished. The renal disease has pushed the patient’s family into severe financial and health crisis.
3. A twenty-three year old tailor (monthly income US$ 65) was diagnosed with ESRD. His parents sold their ancestral home and came to our centre (250 km from his hometown). His applications for grants from the state and central government were rejected for unknown reasons. He could not be accommodated for dialysis in our center and had to continue HD in a private facility. He required multiple hospitalizations for infections. During this process he developed transaminitis, and investigations revealed evidence of hepatitis B and C infections. Lamuvidine was started for hepatitis B, but the patient could not afford treatment for hepatitis C. The transplant had to be delayed because of transaminitis. During this period, the family had to move out of their rented room to free lodgings in a temple. By the time the transaminitis settled, they were left with only US$ 80, which was utilized on the day of transplantation. Post transplant, he was inducted into a drug trial, which enabled him to get immunosuppressive drugs for 6 months. Subsequently, tacrolimus and azathioprine were provided free of cost by a generic pharma company.
4. A twenty-eight old farmer had swelling of lower limbs, which went uninvestigated for 2 years before he was diagnosed with CKD in a private hospital. He soon progressed to ESRD and was initiated on dialysis. He developed catheter related septicemia and required hospitalization. Upon stabilization, he was referred to s for renal transplant. The family sold their ancestral house and Jeep, valued at about US$ 20,200 for US$ 8,100. While on dialysis in a private facility, he was found to have pulmonary tuberculosis, and developed health care associated pneumonia requiring ventilatory support. He also developed critical illness neuropathy and required a long period of convalescence. All the money they had come with was exhausted. At this time he had to borrow money from multiple lenders at high interest rates. Finally he underwent kidney transplant. Free post-transplant immunosuppression was provided for one month on compassionate grounds by a pharmaceutical company. He continues to receive medicines at discounted rates.
5. A thirty-eight year-old male carpenter (monthly income US$ 120) presented to a private hospital with constitutional symptoms. He was admitted, investigated and diagnosed to have ESRD. He spent all his savings (approx. Rs US$ 2,500) in this process. His family included a wife (teacher, monthly income US$ 40) and 4 children (3 sons, 17, 14, 10 and 6 yrs old). He sold his ancestral house for about US$ 5,200 and came to our centre for renal transplant. He had no suitable living donor, and continued on hemodialysis for 13 months at a private dialysis unit, which led to exhaustion of all his finances. Finally, he got a compatible donor as part of swap with another pair. At this time, the family managed to raise US$ 2,000 from houses in their neighborhood, and US$ 2,000 from his landlord at 100% interest. He successfully underwent renal transplantation. However, his wife lost her job as she had exceeded her leave period, and the children dropped out of school. Two of the male children were forced to work as child laborers to make ends meet.
